# Induced pluripotent stem cell treatment for microglia replacement in progressive MS

**DOI:** 10.1177/13524585261459847

**Published:** 2026-07-21

**Authors:** Agastya Reddy Malladi, Naghmeh Abbasi Kasbi, Jeffrey A Cohen, Olaf Stuve

**Affiliations:** Department of Neurology, University of Texas Southwestern Medical Center, Dallas, TX, USA; Department of Neurology, University of Texas Southwestern Medical Center, Dallas, TX, USA; Mellen Center, Neurological Institute, Cleveland Clinic, Cleveland, OH, USA; Department of Neurology, University of Texas Southwestern Medical Center, Dallas, TX, USA; Neurology Section, VA North Texas Health Care System, Dallas, TX, USA; Peter O’Donnell Jr. Brain Institute, University of Texas Southwestern Medical Center, Dallas, TX, USA

**Keywords:** Multiple sclerosis, induced pluripotent stem cells, microglia, stem cell transplantation, progressive multiple sclerosis

## Abstract

**Background:**

Induced pluripotent stem cells (iPSCs) offer a novel approach to target microglia, key drivers of multiple sclerosis (MS) pathology. A promising therapeutic strategy involves depleting resident microglia using colony-stimulating factor 1 receptor (CSF-1R) inhibitors, followed by differentiating iPSCs into microglia in vitro and transplanting them into the central nervous system (CNS). However, no studies have yet specifically examined iPSC-derived microglial replacement in MS.

**Objectives:**

This review evaluates the feasibility of replacing pathogenic microglia in MS with iPSC-derived microglia.

**Methods:**

A systematic literature search identified studies on microglial depletion, iPSC differentiation into microglia, and transplantation, as well as reports involving other MS-relevant neural cell types.

**Results:**

Direct studies on iPSC-derived microglia transplantation in MS are currently lacking; however, several preclinical investigations using iPSC-derived neural stem cells and oligodendrocyte precursor cells in animal models demonstrate robust engraftment, targeted migration to demyelinated lesions, reduced inflammation, enhanced remyelination, and significant functional recovery in vivo. These findings, besides microglia-specific transplantation, depletion, and repopulation studies in other disease contexts, support the biological plausibility of iPSC-based CNS cell replacement and inform future microglial transplantation research.

**Conclusion:**

Replacing pathogenic microglia with iPSC-derived counterparts represents an emerging therapeutic avenue for progressive MS. Rigorous preclinical studies are needed to confirm safety, optimize transplantation, and evaluate long-term efficacy.

## Introduction

Multiple sclerosis (MS) is a chronic, immune-mediated, inflammatory, demyelinating, and neurodegenerative disorder of the central nervous system (CNS)^1^ with neurological disability accumulates from an incomplete recovery from disease relapses and pathogenic mechanisms independent of relapses.^2^

Currently approved disease-modifying therapies (DMTs) for MS are insufficiently effective in countering MS progression. These therapies reduce the frequency of clinical relapses, but ongoing inflammation within the brain and spinal cord, thought to be partly driven by microglia, appears to be insufficiently affected.

In MS, microglia, the resident immune cells of the CNS, hold multiple roles in disease progression and potential recovery. Microglia play a beneficial role in MS recovery by promoting remyelination through their ability to clear myelin debris, secrete growth factors for oligodendrocyte precursor cells (OPCs), and modulate inflammation.^3^ Microglia are also thought to contribute to the demise of oligodendrocytes in MS, through the expression of cytokines and other soluble inflammatory mediators. Subsequently, microglia contribute to synaptic dysfunction and neuronal degeneration, making them a key therapeutic target. One such treatment involves the use of induced pluripotent stem cells (iPSCs) to replace pathogenic microglia. iPSCs are derived from adult somatic cells that are reprogrammed into a pluripotent state, allowing them to be driven to differentiate into a desired cell type.^4^ Following microglial depletion, in vitro differentiation of iPSCs and subsequent transplantation might promote remyelination, reduce inflammation, and restore lost function in patients. By replacing these dysfunctional microglia, iPSC-based therapies aim to address the root of disease progression.

This review aims to establish a general proposal for clinical trials involving the use of iPSCs for microglial replacement in progressive MS. By analyzing both completed and ongoing studies, the review will address key aspects of trial design, discuss the current challenges, and prospects of iPSC-based therapies for progressive forms of disease.

## MS pathogenesis and current treatment

MS manifestations can be viewed along a spectrum, phenotypically classified into the major disease courses of relapsing–remitting (RRMS), secondary progressive (SPMS), and primary progressive MS (PPMS). MS pathogenesis is driven by immune cell infiltration into the CNS and the release of pro-inflammatory mediators. Th1, Th17 cytokines, B-cell-derived inflammatory factors, and CD8+-mediated oligodendrocyte damage collectively drive tissue injury, forming the basis for targeting T and B cells with current DMTs.^5^

Microglia, the CNS’s resident immune cells, are increasingly recognized as key drivers of MS progression, contributing to synaptic dysfunction and irreversible neurodegeneration in both white and gray matter.^3^ Microglia also exacerbate injury by releasing neurotoxic pro-inflammatory cytokines such as IFN-γ and TNF-α, which impair OPC proliferation and hinder remyelination. Remyelination is further compromised by microglial reduced capacity to phagocytose and clear myelin debris.^3^ Considering the central role of microglia in the pathology of MS, depleting pathogenic microglia and replacing them with developed, properly functioning cells may be a potentially effective avenue for treatment.

Currently, there are no interventions that counter disease MS progression in a meaningful way in individuals without disease activity, except for tolebrutinib, which is under regulatory review and has shown promise in phase 2/3 studies. Tolebrutinib inhibits the enzyme Bruton’s tyrosine kinase (BTK), which is involved in B cells, macrophages, and microglial activation and function, which may be pathogenic in MS.^6^ Most current therapies primarily reduce inflammatory activity and relapses but do not effectively prevent disease progression.

In progressive MS, therapeutic options remain limited; ocrelizumab is currently the only approved DMT for PPMS.^7^ Although treatments like natalizumab are highly effective in reducing relapses in RRMS, they show minimal benefit in progressive disease, particularly SPMS.^8^ These disparities reflect fundamental biological differences, which have driven efforts to develop both anti-inflammatory and regenerative therapeutic approaches. In this context, stem cell-based therapies represent a promising strategy to modulate immune dysregulation while facilitating tissue repair.

## Stem cell therapy

Stem cell therapy has been a topic of significant research in the last 35 years. Two distinct strategies have been pursued. Autologous hematopoietic stem cell transplantation (AHSCT) is a potent anti-inflammatory therapy now in phase 3 trials (ClinicalTrials.gov ID NCT04047628). It is most effective in patients with active RRMS and works as an immune “reset,” in which a patient’s hematopoietic stem cells are collected, conditioning chemotherapy ablates the existing immune repertoire, and the purified stem cells are reinfused to reconstitute a more self-tolerant immune system.^9^ Despite its relatively high success rates, this approach carries substantial risks, including chemotherapy-induced immunosuppression before transplantation and the profound post-procedural leukopenia.^10^

Another stem cell therapy strategy is aimed to be restorative, and it is the main focus of this review, encompassing neural, embryonic, induced pluripotent (iPSC), and mesenchymal stem cell (MSC) approaches currently in early clinical investigation. MSCs are adult multipotent cells that can differentiate into various cell types and are being studied in MS for their ability to counteract pro-inflammatory microglial activity. They exert broad immunomodulatory effects by increasing anti-inflammatory mediators and suppressing pro-inflammatory cytokines. Commonly derived from bone marrow, adipose tissue, placental, and umbilical cord tissues, MSCs are highly proliferative and differentiate into osteoblasts, adipocytes, chondrocytes, hepatocytes, and neurocytes. In MS, they may support neuronal regeneration and limit inflammatory damage by migrating to brain lesions and generating glial cells and neural progenitor cells (NPCs).^11^

iPSCs can generate diverse immune- and repair-related CNS cell types, offering potential to replace cells impaired in MS. Derived from adult somatic cells, iPSCs are reprogrammed into a pluripotent state similar to embryonic stem cells (ESCs).^12^ Animal studies show iPSC-based replacement of MS-affected cells (OPCs, NPCs), summarized in Table 1. iPSC-NPCs improve experimental autoimmune encephalomyelitis (EAE) outcomes after intrathecal delivery by migrating to lesions and reducing axonal damage, demyelination, inflammation, and T-cell infiltration, while preserving white matter, enhancing remyelination, and improving function.^13^ Another study shows that iPSC-NPCs can modulate immune responses by reducing T-cell infiltration, preserving white matter integrity, enhancing remyelination, improving motor function, and reducing disease severity.^14^ iPSC-OPCs consistently promote remyelination across multiple demyelination models.^15^ They differentiate into mature oligodendrocytes expressing myelin basic protein (MBP) and proteolipid protein (PLP), enhance myelin formation, and generate structurally compact myelin in vivo, including in shiverer mice.^16^ Transplanted cells also show strong migratory capacity within white matter and improve survival and functional outcomes, outperforming embryonic-derived OPCs in myelination efficiency.^17–19^ MS patient-derived iPSC-OPCs similarly retain remyelinating ability, although astrocytic differentiation remains limited, indicating environmental influence on lineage fate.^20^ In marmoset EAE, iPSC-based cell delivery reduces demyelination and inflammation, while direct parenchymal administration further enhances remyelination of damaged axons.^21^

**Table 1.** Summary of preclinical studies using iPSC-derived cells in various neurological disease models.

Authors (year)	Cell type	Study model	Administration route	Cellular dose and frequency of administration	Results
Laterza et al.^13^	Mouse iPSC-NPCs	EAE mice	Intrathecal transplantation	1 × 10^6^ cells per mouse diluted in 10 μL; single intrathecal transplantation	- Leukemia inhibitory factor (LIF) secretion reduced Th17-mediated inflammatory responses - Reduced immune cell infiltration - Increased survival and differentiation of OPCs - Reduced paralysis in the treated mice
Zhang et al.^14^	Mouse iPSC-NSCs	EAE mice	Lateral ventricle injection	2 × 10^5^ cells in 10 μL PBS; single intraventricular injection	- Injected cells integrated into the CNS, reducing T-cell infiltration and damage to white matter - Promoted remyelination in white matter - Improved motor function and decreased disease symptom scores
Czepiel et al.^15^	Mouse iPSC-OPCs	Cuprizone	Corpus callosum injection	Four microliters of cell suspension at 25,000 cells/microliters concentration; single corpus callosum injection	- 80% of implanted cells did not survive due to the microglial response - Surviving cells became mature myelin basic protein (MBP)+ oligodendrocytes, contributing to remyelination - No iPSCs present post-differentiation procedure
Pouya et al.^16^	Human iPSC-OPCs	Lysolecithin-induced demyelinated optic chiasm	Optic chiasm injection	Cell-transplanted group (*n*= 8): 200,000 hiPSC-derived OPs in 2 µL medium Vehicle-transplanted group (*n* = 8): received 2 µL medium The control group (*n* = 8) received no cells and no vehicle. Each was a single optic chiasm transplantation	- Implanted cells survived and integrated within the optic chiasm, causing remyelination - The majority of integrated cells were MBP+ or proteolipid protein (PLP)+ - Human iPSC-derived OPCs showed recovery in transplanted rats, particularly in the first week post transplantation
Yang et al.^19^	Rat iOPCs	Shiverer mice	Corpus callosum and cerebellum	Bilateral injection with 100,000 purified rat iOPCs; single transplantation	- Implanted cells differentiate into functional oligodendrocytes in vivo - Groups of MBP+ cells formed at injection sites - Dense myelin sheaths observed
Najm et al.^17^	Mouse iOPCs	Shiverer mice	Spinal cord transplantation	5 × 10^4^ cells were injected into the dorsal white matter; a single injection	- iOPCs integrated the white matter and generated tight MBP+ myelin sheaths on axons - Established myelin showed reconstructed major dense lines
Wang et al.^18^	Human iPCS-OPCs	Shiverer mice	Corpus callosum	Bilaterally in the corpus callosum with a total of 100,000 cells or with 300,000 cells; single injection	- Wide-ranged migration of implanted cells and engraftment in the white matter - Differentiation into astroglia and oligodendrocytes, producing compact myelin sheaths - Notably improved survival of mice
Douvaras et al.^20^	Human iPSC-OPCs	Shiverer mice	Forebrain injection	0.5 × 10^5^ in 1 μL of HBSS was injected; single injection	- Injected cells engrafted into the corpus callosum, differentiating into MBP+ oligodendrocytes, supporting myelination
Thiruvalluvan et al.^21^	Human iPSC-OPCs	Cuprizone mice; EAE mice; EAE marmoset	Corpus callosum injection; ICV; intracerebral injection	PBS at a concentration of 12,500 cells per microliter, and 4 μL of cell suspension was stereotactically injected; single injection	- Cuprizone mice: Injected cells engrafted into the corpus callosum, differentiating into MBP+ oligodendrocytes, supporting myelination - EAE mice: Reduction in EAE scores and decreased demyelination; however, there were no detectable cells at the site of lesions - EAE marmoset: Migration toward lesions and differentiation into MBP+ oligodendrocytes

Considering preclinical data supporting iPSC-based approaches in MS and the central role of microglia in MS progression, there is growing interest in therapeutic strategies that directly target microglia. One such approach involves microglial depletion followed by repopulation with healthy iPSC-derived microglia.

## IPSC-derived microglia for replacement of pathogenic microglia

Microglia represent a compelling therapeutic target in MS, given their central role in disease pathogenesis, particularly as the predominant phagocytic cells in smoldering lesions and their contribution to progression independent of relapse activity (PIRA).^22^ Most DMTs have poor CNS penetration due to large size and low lipophilicity, and although cladribine enters the CNS, its impact is minimal at clinical doses.^22^ Approved spingosine-1-phosphate receptor (S1PR) modulators were seen to have had regulatory impacts on microglia with fingolimod reducing the production of pro-inflammatory cytokines in the cuprizone model and siponimod reducing the activity of microglia and macrophages in the EAE model.^23,24^ While complete microglial depletion followed by replacement with iPSC-derived microglia has not yet been studied in MS, iPSCs have been used to generate OPCs and NPCs, underscoring the need to optimize microglial depletion, generation, and CNS delivery. Recent studies in other disease models demonstrate microglial engraftment, repopulation dynamics, and therapeutic effects following transplantation or repopulation (Table 2).^25,26^

**Table 2.** Summary of preclinical studies using iPSC-derived microglia in neurological disease models.

Authors (year)	Cell type	Study model	Microglial depletion strategies	Administration route	Cellular dose and frequency of administration	Results
Abud et al.^27^	Human iPSC-derived microglia-like cells (iMGLs)	MITRG and Rag5xfAD	N/A	Intracranial stereotaxic transplantation into the hippocampus and cortex	N/A	- iMGLs exhibit canonical microglial gene signatures (e.g., P2RY12, APOE, GPR34, and TREM2) - They functionally display key microglial features, including cytokine responses, ADP-dependent chemotaxis, calcium signaling, and efficient complement-mediated phagocytosis - Cells respond appropriately to inflammatory stimuli with immune activation - Successful engraftment in the mouse brain with survival and integration
Mancuso et al.^25^	Human ESC-derived microglia	Immunocompromised mice (Rag2-/- Il2rγ-/- hCSF1KI mice)	BLZ945 CSF1R inhibitor	Intracerebral (bilateral injection into neonatal mouse brain at P4; later intracerebral Aβ injection)	~100,000 cells/µL; 1 µL injected per hemisphere; single transplantation	- Engrafted human microglia-like cells survived and integrated into the mouse brain, taking on typical ramified microglial morphology and expression of homeostatic markers, including Iba1 and TMEM119.
Hasselmann et al.^28^	Human iPSC-derived microglia	MITRG and 5xFAD-MITRG mouse models	N/A	Neonatal: intracerebroventricular transplantation (P0–P1) Adult: intracranial stereotaxic injections (cortex and hippocampus)	Neonatal: ~500,000 cells per mouse (62,500 cells/µL at eight injection sites) Adult: ~2 µL per site at ~50,000 cells/µL (bilateral injections)	- There was robust engraftment and widespread distribution of microglia; cells differentiated into mature microglia expressing markers including P2RY12, TMEM119, and Iba1 - Long-term survival and maturation depend on human CSF1–CSF1R signaling; cells adopt region-specific identities - Transcriptomic profiles closely match ex vivo human microglia, including reactivation of homeostatic gene programs - Functional responses include process motility, injury sensing, and activation following inflammatory challenge - In AD models, cells localize to β-amyloid plaques, adopt DAM-like states, and TREM2 mutants show impaired plaque recruitment
Xu et al.^29^	Human iPSC- and hESC-derived primitive macrophage progenitors (PMPs)	Rag2-/- hCSF1 mice with cuprizone-induced demyelination model	N/A	Neonatal intracerebral transplantation (stereotaxic injections)	~100,000 cells/µL; 0.5 µL per site across four sites (~200,000 cells total per mouse); single transplantation	- Efficient engraftment and widespread distribution across brain regions with mature, ramified microglial morphology - Differentiation into canonical microglia expressing TMEM119, P2RY12, and CX3CR1, with some cells adopting macrophage-like identities - Transcriptomic profiles closely resemble adult human microglia, including upregulation of genes including SALL1 and TREM2 - Functionally, these microglia participated in synaptic pruning and phagocytosis of neuronal and myelin debris - In the cuprizone model, cells respond to injury by clearing myelin and adopting disease-associated activation states
Chadarevian et al.^26^	Human iPSC-derived microglia	MITRG, 5xFAD-MITRG, and hFIRE mice and cuprizone model	PLX3397 CSF1R inhibitor	Intracerebroventricular (neonatal) and intracranial (adult; hippocampus and cortex stereotaxic injections)	Neonatal: ~500,000 cells per mouse (62,500 cells/µL, multiple injection sites) Adult: ~2 µL per site at 62,500 cells/µL (bilateral injections) Both were single transplants	- The transplanted iPSC-derived progenitors engrafted and differentiated in the brain, showing human microglial identity. - Neonatal intracerebroventricular delivery of iPSC-derived progenitors enabled early and widespread brain integration - Stereotaxic intracranial injection in adult mice resulted in more regionally restricted engraftment - Findings suggest iMG can sense and respond to disease-specific cues and adapt their functional profile in vivo

### Microglial depletion strategies

Microglial depletion refers to the complete elimination of pathogenic microglia with a therapeutic intervention. Studies previously demonstrated that the efficiency of microglia transplantation in microglia-containing brains, the actual clinical scenario, is rather low (~0.03%). Currently, the greatest interest in the topic comes with the idea of inhibiting colony-stimulating factor 1 receptors (CSF-1R). CSF-1 is responsible for the development, proliferation, and survival of microglia^30^ through its two main ligands, CSF-1 and interleukin (IL)-34. CSF-1R is a type III receptor tyrosine kinase that undergoes dimerization and autophosphorylation following ligand engagement, activating downstream signaling pathways that drive gene transcription and microglial proliferation.^30^

Three main inhibitors have shown promise in diminishing CSF-1R’s ability to undergo the signaling cascade: PLX3397, PLX5622, and GW2580. Each of these inhibitors possesses the ability to cross the blood–brain barrier (BBB) and selectively inhibit CSF-1R without interfering with the signaling processes of other tyrosine-kinases. PLX3397 (pexidartinib), a selective inhibitor, has been shown to reduce microglial populations by 80.2% after 1 week,^31^ whereasPLX5622 eliminated 90% of microglia within 5 days, demonstrating its potency and rapid CNS bioavailability.^32^ GW2580 is a competitive inhibitor that prevents the binding of adenosine triphosphate (ATP) to CSF-1R, blocking the differentiation pathway and reducing microglial proliferation, additionally decreasing tumor necrosis factor (TNF) production and reducing Iba1 expression in certain neuroinflammatory models.^33^

Partial depletion of host microglia using CSF1R inhibitors creates a permissive niche for donor cell engraftment.^25,26,34^ However, complete depletion might pose significant risks due to microglia’s roles in CNS homeostasis, including debris clearance and immune regulation, and may lead to apoptotic cell accumulation and increased inflammation. Limited human data on CSF1R inhibitors such as pexidartinib suggest feasibility but highlight safety concerns, including hepatotoxicity and systemic immune effects.^35^ Risk mitigation strategies include low-dose or transient depletion before transplantation to preserve immune function while enabling engraftment. CSF1R inhibition is not fully microglia-specific and can affect peripheral macrophages; agents like PLX3397 also inhibit kinases such as c-kit, impacting hematopoiesis.^31^ These off-target effects require careful trial design, including optimized dosing, monitoring of peripheral immune cells, and strategies to limit systemic exposure while maintaining CNS efficacy.

### IPSC differentiation into microglia

Before transplantation into the CNS, microglia should first be generated from iPSCs the culture should be expanded in vitro. Most differentiation protocols mimic the developmental origin of microglia through a two-step process involving first the differentiation into hematopoietic progenitor cells (iHPCs) and then the stimulated differentiation of iHPCs to microglia-like cells (iMGLs).^27^ This process follows the yolk sac-derived ontogeny of microglia, a series of growth factors, cytokines, and specific culture conditions, to produce CD43+ iHPCs that subsequently differentiate into microglia in the presence of macrophage colony-stimulating factor (MCSF), IL-34, and TGFb-1.^26,27^ Over time, approximately 38 days, the cells acquire microglial morphology and express transcription factors.

Across multiple studies, these iPSC-derived microglia exhibit canonical gene signatures (e.g. P2RY12, APOE, GPR34, and TREM2) and functionally display key microglial features, including cytokine responses, ADP-dependent chemotaxis, calcium signaling, and efficient complement-mediated phagocytosis.^27^ The model developed by Abud et al. may be particularly applicable for this therapeutic approach, as it yields iMGLs that more closely resemble primary human microglia and provides a more robust and scalable differentiation platform.

Alternative approaches, such as transcription factor-mediated reprogramming, may reduce differentiation time but can result in cells with less complete microglial identity.^36^ There is an environmental dependency of microglial identity. CNS-derived cues, including TGF-β, CX3CL1, and CD200, are essential for maintaining homeostatic transcriptional states, while co-culture with neurons and brain organoids enhances maturation and induces more in vivo-like microglial features.

In vivo studies demonstrate that transplanted iPSC-derived progenitors can distribute across multiple brain regions and adopt region-specific phenotypes. These cells exhibit transcriptional profiles that closely resemble human microglia with enrichment of core microglial genes such as CSF1R, P2RY12, TMEM119, MERTK, PROS1, and C1QA.^37^ They also display dynamic process motility, respond to injury with chemotactic behavior, and shift into disease-associated states under specific pathological conditions.^28,37,38^ A key concern is pathological activation of engrafted microglia in the MS environment. This may be mitigated by preconditioning donor cells with immunomodulatory cytokines (IL-4, IL-10, TGF-β) to promote an anti-inflammatory phenotype, using concomitant disease-modifying therapies to control inflammation, and implementing post-transplant monitoring for early detection of adverse phenotypic shifts.

### Transplantation

After expanding microglia to the required quantities in culture, the next step is transplantation. Intracranial injection has been the primary delivery method in murine studies and supports efficient engraftment and integration into CNS tissue.^39^ Following neonatal intracerebral transplantation into immunocompromised mice, donor cells survived, integrated into the brain, and comprised a subset of the microglial population, exhibiting broad distribution, typical ramified morphology, and expression of key homeostatic markers, indicating functional incorporation into the CNS.^25^

Across studies, transplanted microglia demonstrated survival, widespread migration, and integration into the CNS. These cells adopt strong morphologies, express canonical markers such as TMEM119, P2RY12, CX3CR1, and C1QA, and mature into competent microglia that resemble endogenous populations.^34^ In addition to maintaining homeostatic functions, engrafted microglia participate in CNS processes including synaptic pruning, phagocytosis of neuronal and oligodendroglial elements, and immune surveillance.^29^

Transplanted microglia respond dynamically to pathological states by migrating to sites of injury and adopting disease-associated activation states.^26^ They show preserved functionality in disease settings, including the ability to migrate to lesions, clear myelin debris, and modulate immune responses through cytokines. In demyelinating and neurodegenerative models, the engrafted cells adopt environment-dependent activation states, highlighting their responsiveness to CNS pathology.^25,26,28,29^ In a cuprizone-induced demyelination model, engrafted human microglia responded to injury by clearing myelin debris and adopting disease-associated activation states (e.g. CD74 and SPP1 upregulation), indicating functional responsiveness to CNS pathology.^29^

Although intracranial delivery is effective, it carries the risks of tissue injury and immune activation. Alternative strategies, including intranasal delivery of iPSC-derived microglia, were found to enable effective migration into the brain and show therapeutic potential in neurodegenerative models, although further validation is required.^39^

Successful transplantation requires donor microglia to cross the CNS and engraft at levels sufficient to counteract MS-associated inflammation (Figure 1). Standardized outcome measures are essential. The expanded disability status scale (EDSS), despite limitations, remains a widely used disability measure, and PET imaging may enable sensitive tracking of iPSC-derived microglia before MRI-detectable changes.^40^ Certain biomarkers also provide insight into disease activity, with CSF markers like chitinase-3-like protein 1 (CHI3L1) and soluble TREM2 reflecting microglial activation, and plasma neurofilament light chain indicating neuroaxonal injury.^41^ Furthermore, advanced MRI metrics offer sensitive structural correlates of microglial-mediated tissue remodeling.^42^ Ultimately, the optimal imaging and analytical metrics will need to be determined in future transplantation studies.

**Figure 1.**
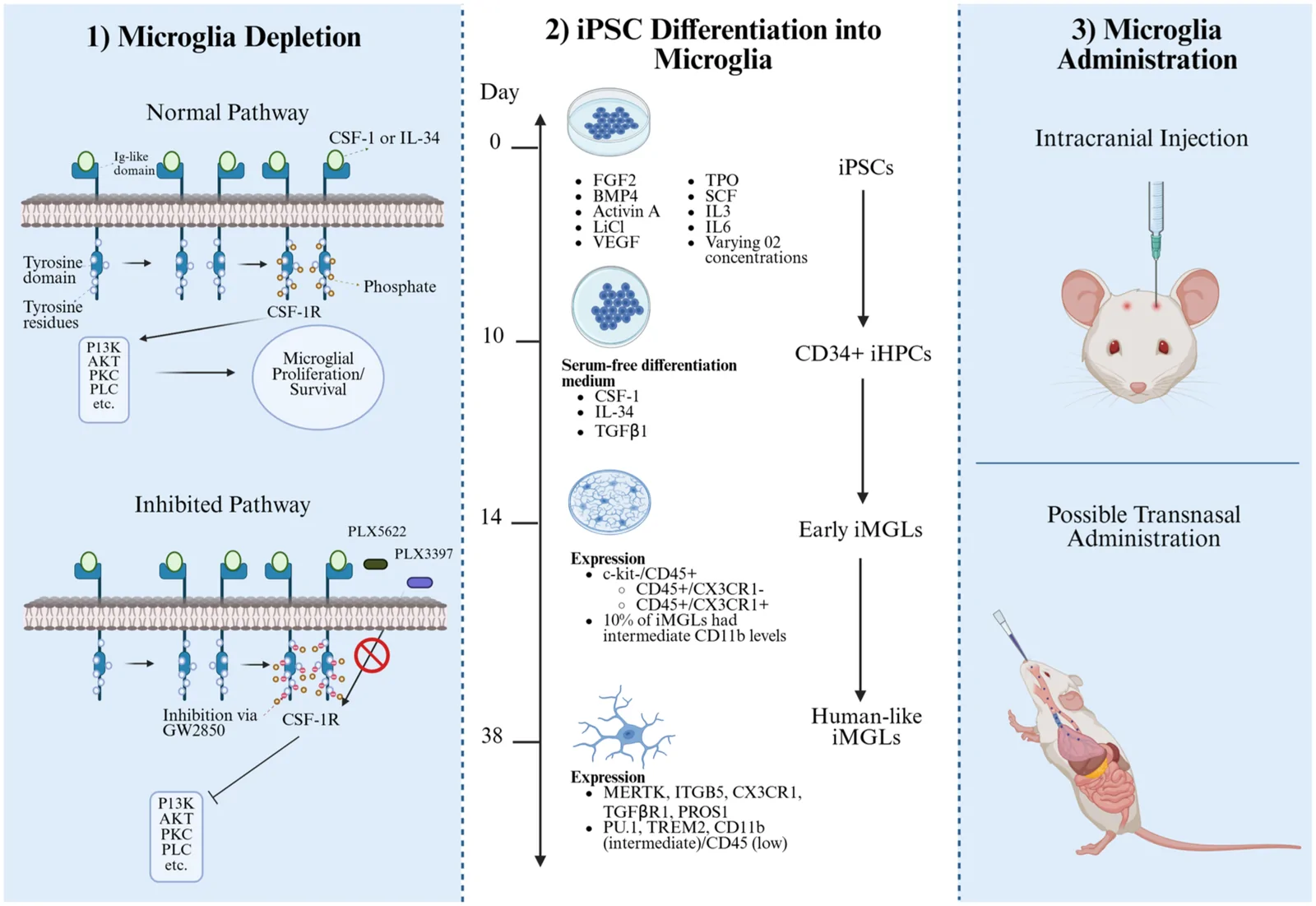
Summary of using iPSC-derived microglia in demyelinating disease models. Microglia, the resident immune cells of the CNS, play a pivotal role in the pathogenesis of multiple sclerosis (MS). Pathogenic microglia contribute to synaptic dysfunction, neuronal degeneration, and the release of pro-inflammatory cytokines such as IFN-γ and TNF-α, which inhibit oligodendrocyte precursor proliferation and impede myelin repair. Their impaired ability to clear myelin debris further limits remyelination. Given their central role in disease progression, strategies aimed at depleting dysfunctional microglia and replacing them with healthy, functional cells hold therapeutic promise. Induced pluripotent stem cell (iPSC)–based approaches may facilitate this by restoring immune balance, promoting remyelination, and enhancing neural recovery (created using Bio Render software).

#### Outlook

MS lacks curative therapies, motivating stem cell approaches. iPSC-derived microglial replacement following depletion of dysfunctional microglia is a promising strategy to target disease progression. Although direct MS data are limited, preclinical models show transplanted microglia can engraft, persist long-term, distribute across the CNS, and perform core functions, reducing neuroinflammation and supporting remyelination. However, engraftment is incomplete, and inflammatory environments may induce maladaptive activation. Key concerns include risks of extensive microglial ablation and off-target effects on peripheral immunity. Future work should prioritize CNS-specific, controlled depletion, alongside optimized delivery, dosing, and monitoring. Overall, this remains an early-stage but promising therapeutic strategy requiring further preclinical validation and rigorous clinical trials to establish safety and efficacy.

## Data Availability

Data sharing is not applicable to this article as no data sets were generated or analyzed during this study.
